# Association of *TP53* rs1042522 G > C, *MDM2* rs2279744 T > G, and *miR-34b/c* rs4938723 T > C polymorphisms with aneuploidy pregnancy susceptibility

**DOI:** 10.1186/s12884-023-05945-3

**Published:** 2023-08-30

**Authors:** Ying Chan, Weiming Xu, Yan Feng, Yan Zhang, Suyun Li, Zibiao Geng, Zhijiao Liu, Qingfen Zhao, Jinman Zhang, Baosheng Zhu

**Affiliations:** 1 Department of Medical Genetics, NHC Key Laboratory of Periconception Health Birth in Western China, Yunnan Provincial Key Laboratory for Birth Defects and Genetic Diseases, First People’s Hospital of Yunnan Province, 157, Jinbi Road, Kunming, 650032 China; 2 Medical Faculty & Affiliated Hospital, Kunming University of Science and Technology, Kunming, Yunnan Province 650500 China; 3 Faculty of Environmental Science and Engineering, Kunming University of Science and Technology, Kunming, Yunnan Province 650500 China

**Keywords:** *TP53* rs1042522 G > C, *MDM2* rs2279744 T > G, *miR-34b/c* rs4938723 T > C, Aneuploidy pregnancy

## Abstract

**Background:**

Aneuploidy pregnancy is a severe major birth defect and causes about 50% spontaneous miscarriages with unknown etiology. To date, only a few epidemiological studies with small sample sizes have investigated the risk factors for aneuploidy pregnancy. *TP53*, *MDM2*, and *miR-34b/c* genes are implicated in tumorigenesis with aneuploidy, yet the function of their polymorphisms in aneuploidy pregnancy susceptibility needs to be clarified.

**Objective:**

To elucidate the association of *TP53* rs1042522 G > C, *MDM2* rs2279744 309 T > G, and *miR-34b/c* rs4938723 T > C specific polymorphisms with aneuploidy pregnancy.

**Methods:**

In the retrospective case-control study, 330 aneuploidies pregnancy women and 813 normal pregnancy controls were recruited between January 2018 and April 2022 at the First People’s Hospital of Yunnan Province, Kunming, China. Three functional polymorphisms, the *TP53* rs1042522 G > C (Arg72Pro), *MDM2* rs2279744 309 T > G, and *miR-34b/c* rs4938723 T > C, were genotyped using the snapshot method.

**Results:**

The frequency distribution of three genotypic variants was not different between case and control pregnant women and was similar to with Hardy-Weinberg Equilibrium (HWE). However, in the younger subgroup (less than 35 years old), a significant difference was detected in allele and recessive model (*p* = 0.01). In the advanced age subgroup (more than or equal to 35 years old), G of *MDM2* rs2279744 T > G revealed a significantly higher frequency in cases than controls (*p* = 0.045), and *miR-34b/c* rs4938723 T > C revealed a significant difference under the dominant model (*p* = 0.03), but no significant differences were observed in other models and in both younger and older subgroup (*p* > 0.05, respectively). These results suggest that individual polymorphisms were not associated with aneuploidy pregnancy, combined with age, they may serve as a risk factor for aneuploidy pregnancy.

**Conclusion:**

Combination of *TP53* rs1042522 G > C, *MDM*2 rs2279744 T > G, and *miR-34*b/c rs4938723 T > C polymorphisms with maternal age may be related to aneuploidy pregnancy susceptibility. These findings might elaborate on the genetic etiology of aneuploidy pregnancy.

**Supplementary Information:**

The online version contains supplementary material available at 10.1186/s12884-023-05945-3.

## Introduction

Embryonic/fetal chromosomal number abnormalities are detected in about 50% of early spontaneous abortions, and aneuploidy is the most common abnormality among the birth defects [1]. However, an accurate understanding of the genetic etiology of aneuploidy pregnancy remains unknown, which could provide valuable information for medical management, reproductive genetic counseling, and supportive patient care [2].Maternal genetic background might partake in the regulation of the formation and continuous development of pregnancy, except for advanced maternal age during the procedure.

*TP53* protein product, p53, is an essential tumor suppressor regulating several fundamental cellular activities, such as cell cycle regulation, senescence, cellular stress, apoptosis, and DNA damage [3]. *TP5*3 gene downregulation triggers somatic chromosomal instability [4]. However, the mechanism underlying this intolerance of nondiploid genomes of cell is yet unknown. A potential role of *TP53* pathway gene mutations was as a risk factor for human aneuploidy; it protects diploid cells and limits the proliferation of aneuploidy in humans [4, 5]. *TP53* plays a role in female reproduction by maintaining germ-cell integrity and various steps of mice and humans [6]. Furthermore, the maintenance of spindle stability during human meiosis and embryonic development is involved in *TP53* [7]. A vital *TP53* gene polymorphism c.215G > C (rs1042522) is related to the accumulation of aneuploidy cells. TP53 rs1042522 G > C is located at codon 72, leading to a transversion from arginine (Arg) to proline (Pro) bringing about the changed structure and function of TP53 [8], which impairs apoptosis [9] and may promotes the continual development of aneuploidy cells [5, 7]. Wild-type *TP*53 is regulated by mouse double minute 2 (MDM2) via a negative feedback regulatory loop, which plays a critical role in the expression level within cells that revealed differential outcomes [10, 11]. *MDM2* rs2279744 T > G polymorphism, deriving of a thymine to guanine in the promoter region for *MDM2* upregulates *MDM2* mRNA and protein levels and affects the TP53 level. Accumulating evidence has shown that TP 53 regulates the expression of various microRNAs [12]. miR-34 family members exhibit critical functions that have been widely studied. In mammals, the miR-34 family consists of three members: miR-34a, miR-34b, and miR-34c. miR-34a is encoded by a unique DNA sequence and miR-34b and miR-34c are derived from the same primary transcript (pri-miR-34b/c) [13]. The common functional polymorphism rs4938723 T > C is located in the promoter of miR-34b/c. Its transition from T to C may alter the binding of the GATA-X transcription factor to the pri-miR-34b/c [14], and its contribution to cancer susceptibility has been illustrated in a few studies [15–18].

Concerning the pivotal roles in cancer, recurrent pregnancy loss susceptibility, and trisomy 21 [16–21], the three important functional nonsynonymous polymorphisms were selected: rs1042522 G > C (Arg72Pro), *MDM2* rs2279744 T > G and rs4938723 T > C. Given the uncertain cause for aneuploidy pregnancy, we conducted a case-control study to evaluate the associations between *TP53* c.215G > C (rs1042522), *MDM2* rs2279744 T > G, and *miR-34b/c* rs4938723 T > C polymorphisms and their interaction with the aneuploidy pregnancy to explore the potential genetic etiology. Therefore, this study would establish a biomarker panel for prenatal prediction and prognosis model of aneuploidy pregnancy women.

## Materials and methods

### Subjects

The case-control study enrolled 1143 women after a prenatal diagnosis procedure, including 330 pregnant women with a fetal chromosome aneuploidy and 813 age-matched normal female controls undergoing prenatal diagnosis in the Department of Medical Genetics at The First People’s Hospital of Yunnan Province, Kunming, China, during January 2018 to April 2022. Written informed consent was obtained before participation in the study. The study protocol is approved by the ethics committees of the First People’s Hospital of Yunnan Province. The study was performed in accordance with the Declaration of Helsinki.

Fetal chromosome abnormality cases were diagnosed as aneuploidy by karyotype analysis and structural abnormality by copy number variation (CNV) method after standard prenatal diagnosis. When one case was diagnosed, 2–3 age-matched normal controls were selected concurrently. Women with an abnormal pregnancy history were excluded from the control group. For the cases, couples with numerical and structural abnormal chromosomes were excluded from the study.

### SNPs selection

Three frequently studied functional polymorphisms of *TP53* rs1042522 G > C, *MDM2* rs2279744 T > G, and *miR-34b/c* rs4938723 T > C were selected in the study for their associations in Down syndrome and some cancer with aneuploidy [7, 15, 16, 19]. *TP53* rs1042522 G > C is located in chr17:7676154 (GRCh38.p14), *MDM2* rs2279744 T > G polymorphism lies in chr12: 68,808,800 (GRCh38.p14), and *miR-34b/c* rs4938723 T > C is situated in chr11:111511840 (GRCh38.p14).

### Genotyping (including DNA extraction)

The residual peripheral blood was collected after the clinical test for genomic DNA extraction using DNA extraction kit (Trelief Hi-Pure Animal Genomic DNA Kit, Qingke), and then DNA was preserved at − 20 ℃ until test. The polymorphisms of *TP53* rs1042522 G > C, *MDM2* rs2279744 T > G, and *miR-34b/c* rs4938723 T > C were tested by SNaPshot SNP typing on a genetic analyzer (ABI 3730xl). The DNA was amplified by polymerase chain reaction (PCR) on a thermal cycler (LongGene A300). The SNaPshot PCR primers are listed in Table S1. The 5-µL reaction consisted of mix 2 µL, SNaPshot PCR primer1 µL, purified PCR products1 µL, and ddH_2_O 1 µL. The amplification in single-base extension system was according to the following conditions: initial denaturation at 96 ℃ for 1 min, 25 cycles of denaturation at 96 ℃ for 10 s, annealing at 50 ℃ for 5 s, and extension at 60 ℃ for 30 s, and maintained at 4 ℃. The electrophoresis sample reaction volume was 11 µL (SNaPshot PCR products:1 µL, Hi-Di formamide: 9.90 µL, and GeneScan LIZ-120: 0.10 µL); the reaction condition was as follows: denaturation at 95 ℃ for 5 min and rapid cooling at − 20 ℃ for 5 min. After spectrum calibration, the samples were separated via capillary electrophoresis. The draft data were analyzed using Gene Mapper software v4.1. The final genotypes were confirmed by Sanger sequencing for 20% repeated sampling and the results were completely in accordance with the SNaPshot.

### Sample size calculation

PASS.11 was used to calculated the sample size. According to the frequencies of alleles analyzed in this study, *TP53* rs1042522 G > C, *MDM2* rs2279744 T > G, and *miR-34b/c* rs4938723 T > C, to set a 95% confidence interval (CI), odds ratios (ORs) as 1.6 based on the published reports, and a 5% margin of error, when the cases reached 330 and controls reached 813; the power of association can be achieved 0.92, then we stopped the sample collection and carried out genotyping.

### Statistical analysis

A chi-squared test was used to test Hardy–Weinberg equilibrium (HWE) for all polymorphisms individually in cases and controls. The differences in continuous variables and nominal variables between case-control groups were compared using a Fisher’s t-test and a two-sided chi-squared test, respectively. Binary logistic regression analysis was used to calculate ORs and 95% CIs. The association of three polymorphisms with aneuploidy pregnancy risk was assessed using ORs and 95% CIs, adjusting by significant difference variables of prenatal diagnosis pregnancy week and previous delivery times. Stratification analysis by age was also conducted to examine whether the effects of *TP53* rs1042522 G > C, *MDM2* rs2279744 T > G, and miR-34b/c rs4938723 T > C polymorphisms were heterogeneous. *p* < 0.05 indicated a statistically significant difference. All statistical analyses were carried out using SPSS version 19.0 (SPSS Inc., IL, USA).

## Results

### Demographic characteristics

The demographic characteristics of fetal aneuploidy pregnant women and normal controls are listed in supporting information Table 1. The differences between cases and controls were assessed for mean age (*p* = 0.48), body mass index (BMI) (*p* = 0.13), prenatal diagnosis pregnancy week (*p* = 0.046), pregnancy times (*p* = 0.43), previous delivery times (*p* = 0.05), nationality (*p* = 0.37), and aneuploidy prenatal diagnosis results.

**Table 1:** Baseline characteristics of study participants

Items	Controls (n = 813)	Cases (n = 330)	*p*
Age (years)	32.41 ± 5.99	32.68 ± 6.29	0.48
BMI	21.46 ± 2.97	21.48 ± 3.11	0.13
Prenatal diagnosis pregnancy week (weeks)	20.27 ± 2.49	20.64 ± 2.89	0.046
Pregnancy times	2.77 ± 1.45	2.85 ± 1.46	0.43
Delivery times	0.71 ± 0.58	0.78 ± 0.66	0.05
Nationality			0.75
Han	623 (76.6%)	231 (71.7%)	0.37^*^
Bai	72 (8.8%)	33 (10.2%)	
Yi	31 (3.8%)	11 (3.4%)	
Hui	19 (2.3%)	7 (2.2) %	
Dai	15 (1.8%)	10 (3.1) %	
Others	53 (6.5%)	30 (9.3) %	
Prenatal diagnosis results			
Trisomy 21		209 (63.3%)	
Trisomy 18		25 (7.5%)	
Trisomy 13		17 (5.1%)	
Sex chromosome aneuploidy		79 (23.9%)	

* The cutoff of p value was 0.003 (0.05/15) after the Bonferroni correction

### **Association of *TP53 *rs1042522 G > C**, ***MDM2 *rs2279744 T > G, and *miR-34b/c *rs4938723 T > C polymorphisms with fetal aneuploidy**

The genotype frequencies and the *p*-values of HWE of *TP53* rs1042522 G > C, *MDM2* rs2279744 T > G, and *miR-34b/c* rs4938723 T > C are shown in Table 2. Neither of the three polymorphisms revealed a significant bias in the HWE in cases (*p* = 0.60 for *TP53* rs1042522 G > C, *p* = 0.42 for *MDM2* rs2279744 T > G, and *p* = 0.16 for *miR-34b/c* rs4938723 T > C) and controls (*p* = 0.86 for *TP53* rs1042522 G > C, *p* = 0.16 for *MDM2* rs2279744 T > G, and *p* = 0.89 for *miR-34b/c* rs4938723 T > C).

**Table 2:** Analysis of alleles, genotypes, and genetic models of *TP53* rs1042522, *MDM2* rs2279744, and *miR-34b/c* rs4938723 between cases and controls

*TP53* rs1042522 G > C						Controls (n = 813)				Cases (n = 330)				OR (95% CI)				*p*			
Genotype			GG			156		19.20%		55	16.70%							0.57^*^			
			GC			403		49.60%		165	50.00%										
			CC			254		31.20%		110	33.30%										
Allele			G			715		43.97%		275	41.67%			1							
			C			911		56.03%		385	58.33%			1.10 (0.92–1.32)				0.31			
Dominant			GG vs. CC + GC											0.84 (0.60–1.18)				0.32			
Recessive			GG + GC vs. CC											0.91 (0,69–1.19)				0.49			
HWE						0.86				0.60											
*MDM2* rs2279744 T > G																					
Genotype			TT			168		20.70%		65	19.70%							0.87^*^			
			TG			425		52.30%		171	51.80%										
			GG			220		27.10%		94	28.50%										
Allele			T			761		46.80%		301	45.61%			1							
			G			865		53.20%		359	54.39%			1.05 (0.88–1.26)				0.60			
Dominant			TT vs. GG + GT											0.94 (0.68–1.29)				0.71			
Recessive			TT + GT vs. GG											0.93 (0.70–1.24)				0.63			
HWE						0.16				0.42											
*miR-34b/c* rs4938723 T > C																					
Genotype			TT			374		46.00%		163	49.40%							0.39^*^			
			TC			356		43.80%		130	39.40%										
			CC			83		10.20%		37	11.20%										
Allele			T			1104		67.90%		456	69.09%			1							
			C			522		32.10%		204	30.91%			0.95 (0.78–1.15)				0.58			
Dominant			TT vs. CC + TC											1.15 (0.89–1.48)				0.29			
Recessive			TT + TC vs. CC											1.11 (0.74–1.67)				0.62			
HWE						0.89				0.16											

*p*-values are adjusted by prenatal diagnosis pregnancy week and previous delivery times.

* The cutoff of p value was 0.166 (0.05/3) after the Bonferroni correction.

The genotype frequency of *TP53* rs1042522 G > C, *MDM2* rs2279744 T > G and miR-34b/c rs4938723 T > C in 330 cases and 813 normal control. None of these polymorphisms showed any significant association between case and control groups in our study population. Moreover, no significant association was observed between *TP53* rs1042522 G > C, *MDM2* rs2279744 T > G, and *miR-34b/c* rs4938723 T > C and fetal aneuploidy pregnancy risk under the dominant, recessive model, and allele analysis.

### **Age-based stratification analysis of *TP53 *rs1042522 G > C**, ***MDM2 *rs2279744 T > G, and *miR-34b/c *rs4938723 T > C polymorphisms with fetal aneuploidy**

According to the significant factor risk of advanced maternal age, the participants were divided into two subgroups stratified by age 35 years. The results of the stratification analysis are summarized in Table 3. In the younger group, the C allele frequency was significantly higher in cases than controls (OR 1.25, 95% CI: 1.03–1.51, *p* = 0.03); a significant difference was detected found in the recessive model (GG + GC vs. CC: OR 1.54, 95% CI: 1.10–2.16, *p* = 0.01). For the older group, a significant difference was found in the recessive model (GG + GC vs. CC: OR 0.58, 95% CI: 0.35–0.94, *p* = 0.03). In the advanced age group, the allele G of *MDM2* rs2279744 T > G revealed a significantly higher frequency distribution in cases than controls (G vs. T: OR 1.30, 95% CI: 1.00–1.68, *p* = 0.045); *miR-34b/c* rs4938723 T > C revealed a significant difference in the dominant model (TT vs. CC + TC: OR 1.61, 95% CI: 1.05–2.46, *p* = 0.03), but no significant differences were found in the other models and in the younger and older subgroup (*p* > 0.05, respectively).

**Table 3:** Stratification analysis of alleles, genotypes, and genetic models of *TP53* rs1042522, *MDM2* rs2279744, and *miR-34b/c* rs4938723 between cases and controls

*TP53* rs1042522 G > C		< 35-years-old											≥ 35-years-old								
		Controls (n = 533)			Cases (n = 206)				OR (95% CI)			*p*	Controls (n = 280)			Cases (n = 124)			OR (95% CI)		*p*
Genotype	GG	112		21.00%	31		15.00%					0.02^*^	44		15.70%	24	19.40%				0.08^*^
	GC	261		49.00%	93		45.10%						142		50.70%	72	58.10%				
	CC	160		30.00%	82		39.80%						94		33.60%	28	22.60%				
Allele	G	485		45.50%	155		37.62%		1				230		41.07%	120	48.39%		1		
	C	581		54.50%	257		62.38%		1.25 (1.03–1.51)			0.03	330		58.93%	128	51.61%		0.85 (0.66–1.10)		0.23
Dominant	GG vs. CC + GC								0.67 (0.43–1.03)			0.07							1.29 (0.74–2.23)		0.37
Recessive	GG + GC vs. CC								1.54 (1.10–2.16)			0.01							0.58 (0.35–0.94)		0.03
*MDM2* rs2279744 T > G																					
Genotype	TT	100		18.80%	44		21.40%					0.55	68		24.30%	21	16.90%				0.24^*^
	TG	292		54.80%	104		50.50%						133		47.50%	67	54.00%				
	GG	141		26.50%	58		28.20%						79		28.20%	36	29.00%				
Allele	T	492		46.15%	192		46.60%		1				269		48.04%	109	43.95%				
	G	574		53.85%	220		53.40%		0.88 (0.73–1.07)			0.20	291		51.96%	139	56.05%		1.30 (1.00–1.68)		0.045
Dominant	TT vs. GG + GT								1.18 (0.79–1.75)			0.42							0.64 (0.37–1.09)		0.102
Recessive	TT + GT vs. GG								1.09 (0.76–1.56)			0.64							1.04 (0.65–1.66)		0.87
*miR-34b/c* rs4938723 T > C																					
Genotype	TT	249		46.70%	93		45.10%					0.92	125		44.60%	70	56.50%				0.02^*^
	TC	227		42.60%	91		44.20%						129		46.10%	39	31.50%				
	CC	57		10.70%	22		10.70%						26		9.30%	15	12.10%				
Allele	T	725		68.01%	277		67.23%		1				379		67.68%	179	72.18%		1		
	C	341		31.99%	135		32.77%		1.04 (0.84–1.27)			0.74	181		32.32%	69	27.82%		0.94 (0.72–1.24)		0.68
Dominant	TT vs. CC + TC								0.94 (0.68–1.30)			0.89							1.61 (1.05–2.46)		0.03
Recessive	TT + TC vs. CC								0.99 (0.59–1.68)			0.99							1.34 (0.69–2.64)		0.39

*p*-values were adjusted by prenatal diagnosis pregnancy week and previous delivery times.

* The cutoff of p value was 0.166(0.05/3) after the Bonferroni correction.

## Discussion

This case-control study explored the association of maternal *TP53* rs1042522 G > C, *MDM2* rs2279744 T > G, and *miR-34b/c* rs4938723 T > C polymorphisms with fetal chromosome aneuploidy. Our preliminary study demonstrated that maternal polymorphisms of *TP53* rs1042522 G > C, *MDM2* rs2279744 T > G, and *miR-34b/c* rs4938723 T > C were not significantly related to the risk of fetal aneuploidy in Chinese population. However, in the age stratification analysis, the frequency of C allele and CC genotype of *TP53* rs1042522 G > C was increased in cases but was decreased in advanced-age women, suggesting its role in fetal chromosome numerical abnormality at different ages.

The clinical significance of *TP53* rs1042522 G > C polymorphism is uncertain at present, regarding the functions of missense, R72 is more efficient than P72 in inducing apoptosis. The dysfunction of this polymorphism might decrease the apoptotic regulation, which would eliminate chromosome numerical abnormality embryos or increase the tolerance to these chromosome instabilities in younger women with *TP53* C allele carriers [4]. *TP53* rs1042522 G > C has also been associated with fetal trisomy 21. C allele and CC genotype are common in the mother with trisomy 21 offspring [5, 19]. This finding was in line with the results in the younger subgroup of our study but contrary to those in the advanced maternal age group perhaps for the coeffect with other environmental factors.

As a benign clinical significance, *MDM2* rs2279744 T > G polymorphism did not show a significant difference between case and control, but G allele is frequent in the advanced age cases subgroup. The result was similar to Salemi et al.; *MDM2* rs2279744 T > G polymorphism showed no significant association with fetal aneuploidy between the cases and controls [15]. For the initial correlation between *MDM2* and *TP53*, and the latter exhibited a regulatory role in human aneuploidy, human reproduction, and maintenance of spindle stability during gamete meiosis [4, 7, 22]. To date, seldom study has analyzed the *MDM2* rs2279744 T > G polymorphism and fetal aneuploidy. Previous studies have shown a close association of *MDM2* rs2279744 T > G with the human polycystic ovarian syndrome and human reproduction [5, 23]. Strikingly, *MDM2* rs2279744 T > G polymorphism is the key element in maintaining the genomic stability of somatic cells [4, 22], which was not revealed in female gametes and embryos.

The miR-34 family is directly regulated by *TP53* as a transcriptional factor. Some studies demonstrated that TP53 inhibits cell proliferation and growth after upregulating miR-34b/c [24]. *TP53* rs1042522 G > C and *miR-34b/c* rs4938723 T > C were associated with the risk of cancers, such as papillary thyroid carcinoma, primary hepatocellular carcinoma, and neuroblastoma [25–28]. The clinical significance of *miR-34b/c* rs4938723 T > C is not reported in ClinVar database. No significant association was observed between *miR-34b/c* rs4938723 T > C and fetal aneuploidy, except for the advanced-age pregnant women in the dominant model in our study. TT genotype revealed an increased frequency distribution among cases, which might result from the small sample size of the subgroup.

Typically, advanced maternal age is the sole risk factor for DS. However, the molecular mechanism of chromosome non-disjunction is yet unknown. A multifactorial etiology of chromosome non-disjunction was found in meiosis [29]. Thus, the present study aimed to identify the putative risk factors and their association with advanced maternal age during oocyte formation and embryo development. Regarding the role of *TP53* and its regulators *MDM2* and *miR-34b/c* in the maintenance of spindle stability, the functional polymorphisms on these genes were investigated. The findings indicated that maternal *TP53* rs1042522 G > C, *MDM2* rs2279744 T > G, and *miR-34b/c* rs4938723 T > C polymorphisms combined with maternal age modified the susceptibility of chromosome non-disjunction allied to oocyte and embryo.

Nevertheless, the present study has some limitations. (1) In the process of aneuploidy oocyte and embryo development, the interaction of many different gene polymorphisms is not simple. The three selected gene polymorphisms resulted in false-negative outcomes. (2) The small sample size might not be sufficient to evaluate an accurate association. (3) All the enrolled population including the normal controls were all recruited from the prenatal diagnosis pregnant women in accordance with the indication of prenatal diagnosis in China. Thus, some selection bias would affect the results. (4) Lack of functional studies about these polymorphisms limits the explanation of results. (5) The origin of fetal aneuploidy was not determined from either paternal or maternal source. (6) The interaction of genes’ polymorphisms and stratification by aneuploidy type was not analyzed for weak associations and a small sample size.

In summary, the current results revealed that the association among *TP53* rs1042522 G > C, *MDM2* rs2279744 T > G, and *miR-34b/c* rs4938723 T > C polymorphisms with fetal aneuploidy susceptibility was observed in an age-dependent manner. Future studies with a larger sample size, diverse ethnicities, geographic regions, and aneuploidy types populations are essential to confirm the results and comprehensively assess the potential function of *TP53* rs1042522 G > C, *MDM2* rs2279744 T > G, and *miR-34b/c* rs4938723 T > polymorphisms in the risk of fetal aneuploidy pregnancy.

### Electronic supplementary material

Below is the link to the electronic supplementary material.

Supplementary Material 1

## Data Availability

The datasets used and/or analyzed during the current study available from the corresponding author on reasonable request.

## Declarations

### Authors’ information

The authors’ team had been occupied the prevention and control of birth defects for more than twenty years and accumulated abundant clinical and basic research experience, especially on the fetal chromosomal abnormality clinical study. All authors are individually mentioned in the author contribution statement in the manuscript.
