# Supplementary material for: Association of TP53 rs1042522 G > C, MDM2 rs2279744 T > G, and miR-34b/c rs4938723 T > C polymorphisms with aneuploidy pregnancy susceptibility
Source: BMC Pregnancy Childbirth. 2023 Aug 30;23:624. doi: 10.1186/s12884-023-05945-3 (PMC10469955; doi:10.1186/s12884-023-05945-3)
Supplement: Supplementary file 1 — Supplementary Material 1 [file 12884_2023_5945_MOESM1_ESM.doc]

| Table S1. Primer sets of outer PCR | | | | |
| --- | --- | --- | --- | --- |
| No. | Sequence of Primer | Length (bp) | Tm (℃) | Products (bp) |
| rs4938723-F | GCAGAGATCTCCCAGAAGTCC | 21 | 60 | 378 |
| rs4938723-R | CTCTTAGAGGGCCATGCAGT | 20 |
| rs2279744-F | GAGCGGTCACTTTTGGGTCT | 20 | 60 | 372 |
| rs2279744-R | CCAATCCCGCCCAGACTAC | 19 |
| rs1042522-F | AGCTGCCCTGGTAGGTTTTC | 20 | 60 | 237 |
| rs1042522-R | TCACCCATCTACAGTCCCCC | 20 |

| Table S2. Primers of the single base extension | | | |
| --- | --- | --- | --- |
| Loci | Primer sequences | Bases (bp) | Direction of extension |
| rs4938723 | TTTTTTTTTTTTTTTTTTTTTTTTTTTTCTGGGAACCTTCTTTGACCTAT | 50 | forward |
| rs2279744 | TTTTTTTTTTTTTTTTTTTTGGCTGCGGGGCCGCT | 35 | forward |
| rs1042522 | TTTTTTTTTTTTTTTTTTGCCAGAGGCTGCTCCCC | 35 | reward |
